# Probiotic detoxification of heavy metals: functional assessment in simulated intestinal and *ex vivo* models

**DOI:** 10.3389/fmicb.2026.1821114

**Published:** 2026-04-29

**Authors:** Marco Pane, Annachiara De Prisco, Angela Amoruso, Giovanni Deusebio, Massimo Marzorati, Marco Corazzari, Romina Monzani, Valentina Saverio, Peter A. Bron

**Affiliations:** 1 Probiotical Research s.r.l., Novara, Italy; 2 Center for Microbial Ecology and Technology (CMET), Faculty of Bioscience Engineering, Ghent University, Ghent, Belgium; 3 Department of Health Sciences and Center for Translational Research on Autoimmune and Allergic Disease (CAAD), University of Piemonte Orientale, Novara, Italy; 4 Bron Consultancy, Wageningen, Netherlands

**Keywords:** bioremediation, GEVS, heavy metal detoxification, lactobacilli, pollution, probiotic, SHIME

## Abstract

**Introduction:**

Bioremediation is an emerging, sustainable strategy that relies on microbial processes to detoxify environmental pollutants. Among these pollutants, heavy metals (HMs) are pervasive, non-degradable toxic elements that pose serious risks to human health. In this study, we individually evaluated three probiotic lactobacilli strains—*Lactiplantibacillus plantarum* LP14, *Lactobacillus crispatus* LCR04, and *Lactobacillus acidophilus* LA12—for their capacity to detoxify cadmium, chromium, mercury, and lead in the gastrointestinal (GI) tract, as well as their capacity to mitigate heavy metal-induced intestinal damage, with a single-strain product strategy in mind.

**Methods:**

After initial selection of the strains for their *in vitro* detoxifying potential, we employed a dynamic Simulator of the Human Intestinal Microbial Ecosystem (SHIME®) model and each strain’s survival, growth, and heavy metal detoxification capacity was assessed under sequential gastric, small-intestinal, and colonic conditions.

**Results:**

Strain- and metal-specific differences in HMs sequestration emerged: *L. plantarum* LP14 and *L. crispatus* LCR04 showed robust persistence and significantly reduced HMs bioavailability, whereas *L. acidophilus* LA12 displayed minimal detoxification under GI simulation. Mechanistically, only the strains that proliferated in the colonic phase achieved substantial HM removal, underscoring the importance of metabolic activity *in situ*. In a gut *ex vivo* system (GEVS), heavy metal exposure alone compromised epithelial barrier integrity and triggered pro-inflammatory responses. Pre-incubation of the HMs with each of the probiotic strains markedly alleviated these effects, restoring intestinal permeability and cytokine profiles.

**Discussion:**

Overall, the results demonstrate a novel probiotic-based intestinal bioremediation strategy and highlight the translational potential of targeted single-strain probiotic interventions, reducing heavy metal exposure to protect gut health.

## 1 Introduction

Through their domestic, agricultural, medical, industrial, and technological applications, levels of chemical elements above those occurring naturally are nowadays routinely observed in soil, air, water, crops, and animals throughout the planet (Astolfi et al., 2019). Consequently, the general human population is unavoidably exposed to toxic levels of these chemical elements via contaminations in drinking water, as well as the food chain, e.g., fish (Elumalai et al., 2023; Makedonski et al., 2017), vegetables (Manwani et al., 2023), dairy (Scutarașu and Trincă, 2023), and drinking water (Rehman et al., 2018). Among these chemical elements, heavy metals (HMs) are of particular concern to negatively impacting human health. Accordingly, certain HMs like cadmium (Cd), led (Pb), and mercury (Hg), deserve attention as they are classified as probably carcinogenic to humans or carcinogenic to humans based on their chemical form (organic and inorganic) and exposure routes (Astolfi et al., 2019; Hou et al., 2025).

Besides their carcinogenic properties, HMs can perturbate the composition and functionality of the gut microbiota, which plays a pivotal role in the maintenance of intestinal homeostasis (Duan et al., 2020). The exact perturbations are HM-specific but the consistent body of knowledge on the effects of HMs on gut microbiota composition depicts a general indication where the abundance of *Firmicutes* and *Proteobacteria* decreases, while *Bacteroidetes* populations rise, although more profound changes in microbiota composition are reported (Duan et al., 2020; Bist and Choudhary, 2022; Chen et al., 2022). These altered microbiota population dynamics can lead to changes in epithelial barrier integrity, redox status, as well as (the level of) several metabolites (short chain fatty acids, bile salt conjugates, amino acids metabolism-derived compounds, and lipids) (Duan et al., 2020). Concomitant to microbiome alterations, health status can be impacted, e.g., it was demonstrated that Pb exposure in mice led to a decrease of specific gut population such as *Akkermansia* spp. counteracted by an increase in other gut members like *Desulfovibrio* spp., and an associated weight gain and metabolic disorders (Duan et al., 2020). Furthermore, as demonstrated in animal model, Pb exposure during gestation can result in profound alteration of the intestinal microbiota in the offspring and increased bodyweight in male in their adulthood (Wu et al., 2016). By contrast, Chromium (Cr) supplementation (mainly as chromium picolinate and chromium malate) is applied as potential strategy in ameliorating glucose blood levels and metabolic disorders in type 2 diabetic individuals (Barra et al., 2021). This supplementation is demonstrated to also have positive effects on gut microbiota that showed increased levels of Gram (+) bacteria correlated with improved insulin sensitivity and cardiovascular measures (Barra et al., 2021). However, Cr supplementation is still considered controversial due to the potential (geno)toxic effects that it might exert on the general population (Levina and Lay, 2008). Taken together, information, partly from animal studies, on the impact of individual heavy metals on the gut microbiome and health status is available but data on the impact of their combined effects in humans is more scattered.

Interestingly, the relationship between chemical pollutants and gut microbiota can be regarded as bidirectional. Indeed, if on one hand gut microbiota can be severely affected by HMs, it is increasingly acknowledged as a dynamic interface for heavy metal detoxification. This evidence is contributing to the conceptual emergence of ‘intestinal bioremediation’, a probiotic-driven strategy for mitigating toxic metal burden in the host (Monachese et al., 2012; Wu et al., 2017). Several mechanisms have been established and include bacterial HM binding, complexation/chelation, intracellular accumulation, enzymatic/chemical modification [mainly oxidation, reduction, (de)methylation], as well as indirect mechanisms, e.g., by reinforcing the gut barrier function. Besides endogenous microbiota members, probiotic species, including several lactobacilli, have demonstrated bioremediation properties (Abdel-Megeed, 2021; Arun et al., 2021). For instance, supplementation of mothers with a mixture of *Lacticaseibacillus paracasei* DSM 24733, *Lactiplantibacillus plantarum* DSM 24730, *Lactobacillus acidophilus* DSM 24735, *Lactobacillus delbrueckii subsp. bulgaricus* DSM 24734, combined with several bifidobacterial and streptococcal species, resulted in significantly reduced Cd levels in stool samples obtained from their breast-fed newborn (Astolfi et al., 2019). Furthermore, the ability of *L. plantarum* TW1-1 in Cr detoxification was demonstrated in an animal model, where mice co-administered with Cr and *L. plantarum* TW1-1 showed increased fecal levels of Cr^3+^ attributable to a weaker Cr intestinal adsorption (Wu et al., 2017).

For this study, we aimed to assess the ability of different probiotic strains to counteract HMs in a multilevel *in vitro* approach. Unlike previous studies that have typically evaluated single strain-single HM interactions, here we exposed three extensively characterized, gut-derived probiotic lactobacilli—*Lactiplantibacillus plantarum* LP14, *Lactobacillus crispatus* LCR04, and *Lactobacillus acidophilus* LA12—known for their established safety and functional properties to a complex mixture of Cd, Cr, Hg, and Pb, to evaluate both their growth performance and their bioremediation potential under *in vitro* growth and post-growth conditions. To further explore their translational relevance, each strain was assessed in the SHIME® model (Tierney et al., 2023), simulating dynamic gastrointestinal conditions, where strain- and metal-specific detoxification profiles emerged. Finally, their protective effects were validated in an *ex vivo* gut model (Yissachar et al., 2017), indicating pre-incubation with heavy metals significantly attenuated epithelial barrier disruption and inflammatory cytokine induction. This progressive increase in model complexity — from standard *in vitro* conditions to dynamic gastrointestinal simulation and *ex vivo* gut models — represents, to our knowledge, a novel multilevel approach to study probiotic lactobacilli in HM bioremediation, enabling more extensive strain characterization prior to *in vivo* validation. This integrated approach underscores the functional diversity of probiotic lactobacilli in HM bioremediation and supports their targeted application for gut detoxification.

## 2 Materials and methods

### 2.1 Strains and culture conditions for *in vtiro* HM detoxification experiments

*Lactiplantibacillus plantarum* LP14 (DSM 33401), *Lactobacillus acidophilus* LA12 (LMG P-34340), and *Lactobacillus crispatus* LCR04 (DSM 33487) were obtained from the Probiotical S. P. A Master Cell Bank. Bacterial cultures used in this study were activated by inoculating one glycerol stock at 10% in fresh de Man Rogosa Sharpe medium (MRS), supplemented with 0.05% L-cysteine, followed by 24 h-anaerobic incubation at 37 °C. Full-grown bacterial cultures were sub-cultured (2%) in fresh MRS prior to their use in the experiments below. Heavy metals (HMs) included in this study were Cadmium (Cd), Chromium (Cr), Mercury (Hg) and Lead (Pb). HMs were purchased in their inorganic form, namely CdS0_4_ (Sigma Aldrich 481,882), CrCl_3_ (Sigma Aldrich 230,723), Hg(NO_3_)_2_ (Sigma Aldrich 104,439) and PbCl_2_ (Sigma Aldrich 268,690). HMs were mixed to demineralized water to obtain stock solutions. HM solutions were sterilized by 0.22 μm filtration. Final concentrations employed in each of the experiments is detailed in the result sections.

### 2.2 HM quantification

Samples were analysed for heavy metal content by Inductively Coupled Plasma Mass Spectrometry (ICP-MS) after acid digestion. The aqueous sample was transferred to an Erlenmeyer flask and added to concentrated nitric acid (1 mL) and H_2_O_2_ on a hot plate under reflux conditions for 20 min until complete mineralization, which is achieved when the solution is clear and transparent. After cooling to room temperature, samples were transferred to a volumetric calibrated flask and brought to volume with ultrapure water. Calibration standards were matrix-matched to the sample digest solution to minimize matrix-induced signal suppression or enhancement; detailed information on the calibration standards, instrumental conditions, and acquisition parameters for each analyte are provided in the Supplementary Table S1. Samples were then analysed using a Thermo Scientific™ iCAP™ RQ ICP-MS with a RF power of 1,550 W and a collision gas flow of 4–4.6 L/min. All the HM experiments and quantifications were run in triplicate. Results are calculated as reduction (%) of individual HMs by comparing levels obtained before and after HM contact with bacterial cells, with initial HM concentrations in the stock solution also measured as a positive control. Results are expressed as mean values ± standard deviations.

### 2.3 Preliminary *in vitro* assessment of heavy metals detoxification by lactobacilli

Probiotic strains LP14, LA12 and LCR04 were studied for their potential ability to detoxify HMs in a preliminary *in vitro* experiment. Bacterial cultures were inoculated with HM solutions (see Results section for HM concentrations) to start two different challenge tests at the end of which the HM concentrations were quantified as described above. In the first test, growth media with HM solution were prepared, to which bacterial cells were added to an OD_600_ of 0.2, followed by incubation for 24 h at 37 °C under anaerobic conditions (co-culturing test). In the second test, bacterial cultures were first inoculated at an OD_600_ of 0.2 in growth media without HMs, followed by incubation according to times and temperatures reported above to allow bacterial growth. Subsequently, the HM solution was added to the full-grown, overnight bacterial cultures (for 24 h, 37 °C) to favour possible interactions (post-growth test). Experiments were performed in three independent biological replicates for each condition tested.

### 2.4 SHIME set-up

The Simulator of Human Intestinal Microbial Ecosystem (SHIME®) in the configuration for dynamic upper GIT simulation, followed by a short-term colonic simulation under fed condition was used, with modifications to mimic ingestion of an HM-contaminated food matrix.

#### 2.4.1 Upper GIT simulation

The upper GIT simulation was performed in double-jacketed reactors simulating the gastric and small intestinal digestion conditions in a sequential order. The temperature was maintained at 37 °C during the entire upper GIT simulation and magnetic stirring (300 rpm) was applied to homogenize the reactor content. Continuous pH control was implemented by using a Senseline pH meter F410 (ProSense, Oosterhout, The Netherlands) and an automatic pump dosage of HCl (0.5 M; Chem-lab, Zedelgem, Belgium) or NaOH (0.5 M; Chem-lab). To mimic fed conditions (i.e., administration of the test product during or immediately after consumption of a meal), specific pH profiles, enzyme levels, and retention times were set accordingly. In addition, the gastric and small intestinal juices were specifically designed to mimic fed environmental conditions. The stomach digestion had a total duration time of 120 min; during this timeframe the pH decreased from 4.6 to 3.0 in a sigmoidal way by the controlled addition of HCl (0.5 M) at established time points. Pepsin was supplied with the activity being standardized by measuring absorbance increase at 280 nm of trichloroacetic acid -soluble products upon digestion of haemoglobin (reference protein). Gastric simulating juice (222.4 mL) containing the SHIME® nutritional medium (23.64 g/L of product PDNM001B from ProDigest), 4.18 g/L NaCl (VWR, Leuven, Belgium) and 0.76 g/L KCl (Chem-lab) was initially mixed with 1.3 mL lecithin (13.5 g/L; Carl Roth GmbH, Karlsruhe, Germany) and 12.1 mL pepsin (40 g/L; Chem-lab) at the incubation (starting point). The pH of this mixture was adapted to 4.6 prior to the addition of 33.7 mL HM stock solution. After 120 min of gastric incubation, environmental conditions were adapted to mimic small intestinal (SI) conditions. Pancreatic juice (99.6 mL) was constituted by 8.16 g/L NaHCO3 (Chem-lab), 15.9 g/L oxgall (Becton-Dickinson, Erembodegem, Belgium), 10.6 g/L pancreatin (Merck Life Science, Hoeilaart, Belgium), 6.45 mL trypsin solution (10 g/L; Carl Roth GmbH) and 8.1 mL chymotrypsin solution (10 g/L; Carl Roth GmbH). Next, sterile dH2O was added to reach a total volume of 451.05 mL. Environmental pH in this phase increased from 3.0 to 5.5. Cell inoculum was injected in the small intestinal simulator reactor. Lyophilized LP14, LA12 and LCR04 were resuspended in sterile dH2O up to a volume of 6 mL with a standardized cellular concentration to obtain a final cell concentration of 10^10^ cells/reactor. After probiotic acclimatization, samples of each strain were added separately to the reactor (test condition). Similarly, 6 mL of pure sterile dH2O were added to a reactor to simulate the blank condition (no cell inoculum). After sample inoculum the small intestinal incubation was initiated. The environmental pH was increased from 5.5 to 6.5 and maintained at this pH over a 27 min period, simulating the duodenal incubation (DUO). This phase was then followed by a stepwise pH increase (i.e., 0.1 pH units every 7 min) to 7.5 within a 63 min-period, mimicking the jejunal environment (JEJ). Finally, the pH remained constant at 7.5 during 90 min simulating the ileal incubation. The pH increase was achieved by the addition of NaHCO3 solution (4.8 g/L) following 60, 90, and 120 min of small intestinal incubation. As such, the dilution of the intestinal contents was simulated (Riethorst et al., 2018). The entire small intestinal incubation was performed under anaerobic conditions.

#### 2.4.2 Short term colonic tract simulation

At the end of the SI incubation, the colonic simulation (proximal colon) was started in a subsequent reactor. Colonic environment (Colon) was resembled by the addition of 40 mL upper GIT suspension to 86 mL fresh colonic medium constituted by 23.89 g/L KH2PO4 (Chem-lab), 7.61 g/L K2HPO4 (Chem-lab), 2.93 g/L NaHCO3, 2.93 g/L yeast extract (Oxoid, Basingstoke, GB), 2.93 g/L peptone (Oxoid), 1.16 g/L glucose (Merck Millipore, Massachusetts, USA), 2.32 g/L starch (Sourby, Roeselare, Belgium), 1.46 g/L mucin (Carl Roth GmbH + Co. KG), 0.74 g/L L-cysteine HCl (Merck, Overijse, Belgium), and 2.92 mL Tween® 80 (Sigma-Aldrich, Overijse, Belgium) and 14 mL filter-sterilized fecal inoculum. For this specific investigation, a sterile colon environment was simulated. Accordingly, fecal inoculum was derived from a healthy adult human donor and prepared as described earlier by Van den Abbeele, Kamil et al. (2018) and Van den Abbeele, Taminiau et al. (2018). Briefly, a mixture of 1:10 (w/v) of fecal sample and anaerobic phosphate buffer (8.8 g/L K2HPO4, 6.8 g/L KH2PO4, 0.01 g/L sodium thioglycolate) and 0.015 g/L sodium dithionite (Merck) was homogenized for 10 min (BagMixer 400, Interscience, Louvain-La-Neuve, Belgium). After centrifugeation for 2 min at 500 × g (Centrifuge 5417C, Eppendorf, VWR), large particles were removed and the remaining suspension was further processed via two subsequent centrifugation steps (same operative condition as above). The remaining supernatants were finally filtrated in two consecutive steps, using a 0.45 μm bottle-top-filter (VWR) and a subsequent 0.22 μm bottletop-filter (Carl Roth GmbH + Co. KG) in combination with a vacuum pump (N816 3KN, KNF lab, Aartselaar, Belgium) to discard the microbial fraction. Filter-sterilization of the inoculum was performed to allow studying the fate of the supplemented probiotic strains in the colon in terms of viability and HM detoxification. The environmental pH of the colonic incubation at starting point was equal to 6.5. The buffering capacity of the colonic medium maintained the environmental pH between 6.5 and 5.8 during the entire colonic simulation in order to mimic the proximal colon. The entire colonic incubation had a total duration of 24 h and was performed under anaerobic conditions at 37 °C and 90 rpm agitation (MaxQ 4,000 Benchtop Orbital Shaker, Thermo Fisher Scientific, Belgium).

#### 2.4.3 Sample collection

Samples (2 mL) were collected during the upper GIT and short-term colonic simulation at different time points; at the beginning of the stomach incubation (= ST start), at the beginning of the small intestinal incubation (= SI start, immediately after addition of the activated culture and/or sterile dH2O), at the end of the duodenal incubation (= DUO end), at the end of the jejunal incubation (= JEJ end), at the end of the ileal incubation (= ILE end), and at the beginning and at the end of the colonic incubation (Colon = C0h and Colon = C24h), respectively. Samples at abovementioned timepoints were directly analysed for cell enumeration. Additionally, samples were centrifuged (6 min at 6000 rpm) to obtain two fractions, namely cell-free supernatants and cell pellets that were both analysed for determination of the HMs content and distribution.

#### 2.4.4 Cell enumeration

For each sample, a ten-fold dilution series was initially prepared in anaerobic PBS. Cell enumeration of probiotic bacteria was done by staining the appropriate dilutions with SYTO 24 (final concentration of 1 μM; Life Technologies Europe, Merelbeke, Belgium) and propidium iodide (1.33 μM final concentration; Thermo Fisher Scientific, Merelbeke, Belgium) for 15′ at 37° C of incubation in the dark. Samples were analyzed on a BD Accuri C6 Plus (BD Biosciences, Vianen, The Netherlands) using the high flow rate. Bacterial cells were separated from medium debris and signal noise by applying two threshold values, i.e., a primary FSC-H threshold of 500 and a secondary FL-1 threshold of 700. Flow cytometry data were analyzed using FlowJo, version 10.5.2 and results were reported as average log (counts/reactor) ± SD. (*n* = 3). Results were expressed as Active Fluorescent Unit (AFU)/ml taking into account the changes in volume occurring during the experiments through the reactors and so by applying correction factors.

#### 2.4.5 Data analysis

Three independent trials were carried out for each strain. Cell density data measured at the different timepoints are reported as mean values ± standard deviation. Variations in HMs concentration for samples collected at ILE end (end of incubation in small intestinal simulated conditions) and Colon 24 h (end of incubation in colonic simulated conditions) were expressed as relative to the HMs concentration at ST start (beginning of gastric simulated phase). Results were adjusted considering the changes in volume occurring during the experiments through the reactors by applying correction factors (Supplementary Table S2) and are expressed as mean values (*n* = 3) ± standard deviations. A t-test was performed to analyse the differences found in HM concentrations detected in the lactobacilli-HMs co-incubated samples vs. the blank sample (condition where HMs were inoculated in the SHIME reactors without probiotic addition) to determine the statistical significance. Significance was presented in the following categories; 0.01 < *p*-value ≤ 0.05 (*), 0.01 < *p*-value ≤ 0.001 (**) and 0.001 < *p*-value ≤ 0.0001 (***). For each probiotic strain, statistically significant differences between the viable population densities (within the same strain) were determined between each sampling point and its preceding one to demonstrate changes in viable population. Significance was established by t-test; 0.01 < *p*-value ≤ 0.05 (*), 0.01 < *p*-value ≤ 0.001 (**) and 0.001 < *p*-value ≤ 0.0001 (***).

### 2.5 Gut e*x vivo* model for cytokine and epithelial barrier measurements

#### 2.5.1 Gut *ex vivo* system

Small intestines from 13 days-old C57BL/6 J mice were freshly resected and cultivated in a silicone based gut *ex vivo* system (GEVS) with serum free tissue culture medium containing Iscove’s Modified Dulbecco’s Medium (IMDM, Gibco, CA, USA) supplemented with 20% KnockOut serum replacement (Gibco), 2% B-27 and 1% N-2 supplements (Gibco), 1% L glutamine, 1% non-essential amino acids (NEAA) and 1% HEPES (Gibco). GEVS was set for the experiments according to the procedure reported by Gagliardi, Clemente et al. (2021) and Gagliardi, Monzani et al. (2021). Briefly, the model consists of six independent chambers for the insertion of the resected intestines. Each chamber is connected to two needles (input/output syringes) allowing the flow of complete nourishing medium in the inner intestinal compartment (luminal flow; flow rate of 99 μL/h), while tissues are imbibed with complete Iscove’s medium (outer medium) to keep the intestine alive. During the experiments the temperature of GEVS was kept at 37 °C using a standard laboratory warming plate and heat spreader to ensure an optimal and constant heat transfer. A proper tissue oxygenation was ensured by humidified O_2_ (95%) and CO_2_ (5%) gas mixture injection into the device chamber. Ex vivo experiments were performed in two independent biological replicates for each condition. In each experiment, two intestines were used per condition (total *n* = 4 biological samples per condition). This experimental design reflects the intrinsic characteristics of the GEVS platform, which allows multiple conditions to be tested within the same tissue while reducing animal usage.

#### 2.5.2 Preparation of cell free supernatants after co-incubation of probiotics and HM media

Bacterial cultures were all cultivated in MRS medium through incubation at 37 °C for 6–8 h. After incubation, bacterial cell cultures were rechecked for cell enumeration by flow cytometry and cell concentrations for all the strains were attested at 9.0 ± 0.05 log AFU/ml. After bacterial growth, HM stock solution was added in ratio 1:10 to bacterial cultures for overnight (16 h) co-incubation at 37 °C. Samples were then centrifuged (4,000 rpm x 10 min) to collect the cell-free supernatant (CFS) to use in GEVS. Similarly, HM stock solutions were added in ratio 1:10 to MRS medium without bacterial inoculum and incubated overnight at 37 °C.

#### 2.5.3 GEVS treatments

GEVS were stimulated for 5 h with Iscove’s medium alone (control condition, Ctrl), or complete Iscove’s medium added (ratio 1:10) of the HMs recovered after incubation in MRS (MIX) or Iscove’s medium added (ratio 1:10) of CFS recovered after preliminary probiotics-HMs co-incubation (MIX+ CFS LP14, MIX+ CFS LCR04 and MIX+ CFS LA12). Tissue samples were then checked for tissue integrity/permeability and inflammation markers as described below.

#### 2.5.4 Tissue permeability

Tissue permeability was evaluated by measuring FITC-Dextran (FD4; Merck) release into external medium (Bootz-Maoz et al., 2023). Briefly, FITC-Dextran medium was administered with an input syringe connected to each intestinal lumen, at a concentration of 0.1 mg/mL in a final volume of 5 mL, using a stock solution of 100 mg/mL. After stimulation, the outer medium in contact with the intestinal tracts was collected. Fluorescence was measured by spectrophotometric assay (SPARK Multimode Microplate Reader TECAN) in 96-well plates (excitation: 485 nm, emission: 528 nm). Before sample analysis, a calibration curve for FITC-Dextran was obtained by measuring serially diluted FITC-Dextran stock solutions (0, 20, 40, 60, 80, 100 μg/mL). Subsequently, 100 μL of each sample was measured in 96-well microplate and FITC-Dextran concentrations were obtained using standard curve interpolation.

#### 2.5.5 RNA extraction and quantitative real-time PCR (qPCR) cytokine assay

TripleXtractor reagent (Grisp) was used to isolate total RNA from tissues. ExcelRT Reverse Transcription Kit was used to produce cDNA according to the Manufacturer’s recommendations (Grisp). Quantitative PCR reaction was performed on a CFX96 BioRad thermocycler. ExcelTaq™ 2X Fast Q-PCR Master Mix (SMOBIO) was used to produce amplicon products during repetitive cycling and the melting curve protocol was used to check for probe specificity. Primers were designed using the IDT PrimerQuest Tool software (IDT, Integrated DNA Technologies Inc., IA, USA; https://eu.idtdna.com/Primerquest/Home/Index). Primer sequences are reported in Supplementary Table S3. Results were normalized using L34 as internal control and comparative Ct method (ΔΔCt) was used for relative quantification of gene expression. qPCR analyses were performed using technical duplicates for each sample obtained from each biological replicate. Reported values represent the mean ± SD of independent experiments.

#### 2.5.6 Tissue viability

Tissue viability was evaluated through AlamarBlue staining (Thermo). Briefly, tissues were recovered from GEVS, weighed, placed in a 48-well plate, and incubated with DMEM supplemented with 10% FBS, 2 mM L-glutamine, 100 U/mL Penicillin, 0.1 mg/mL Streptomycin (Euroclone) and 100 g/mL AlamarBlue. After 2 h, the absorbance of 100 μL of medium from each sample was analyzed by a SPARK Multimode Microplate Reader (TECAN). Data was normalized by dividing the absorbance by tissue weight. Viability of untreated tissue (CTRL) was set to 100%.

#### 2.5.7 Statistical analysis

All experiments were performed as technical duplicates and repeated twice, while statistical analysis was performed using GraphPad Prism 7. The student t-test or ANOVA were used to determine statistical significance. A *p*-value equal to or less than 0.05 was considered significant. mRNA expression levels were represented as ‘fold change over control’, r.l. relative levels. Histograms represent mean ± SD; **** *p* < 0.0001; *** *p* < 0.001; ** *p* < 0.01; * *p* < 0.05; ns = non-significant.

## 3 Results

### 3.1 Establishing *in vitro* HM detoxification by lactobacilli

*Lactiplantibacillus plantarum* LP14, *Lactobacillus acidophilus* LA12 and *Lactobacillus crispatus* LCR04 were studied for their potential ability to detoxify a mixture of four HMs, namely Cd^2+^ (1.1 mg/L final concentration), Cr^3+^ (17.6 mg/L), Hg^2+^ (8.8 mg/L), and Pb^2+^ (14.9 mg/L) in a two-arm experiment with cells and HMs, either co-incubated during growth or HMs added post-growth. The rationale behind this set-up was that detoxification of HMs by living bacteria can occur via different mechanisms based on cell/HM contact or via an active cell metabolism on HMs.

**Figure 1:**
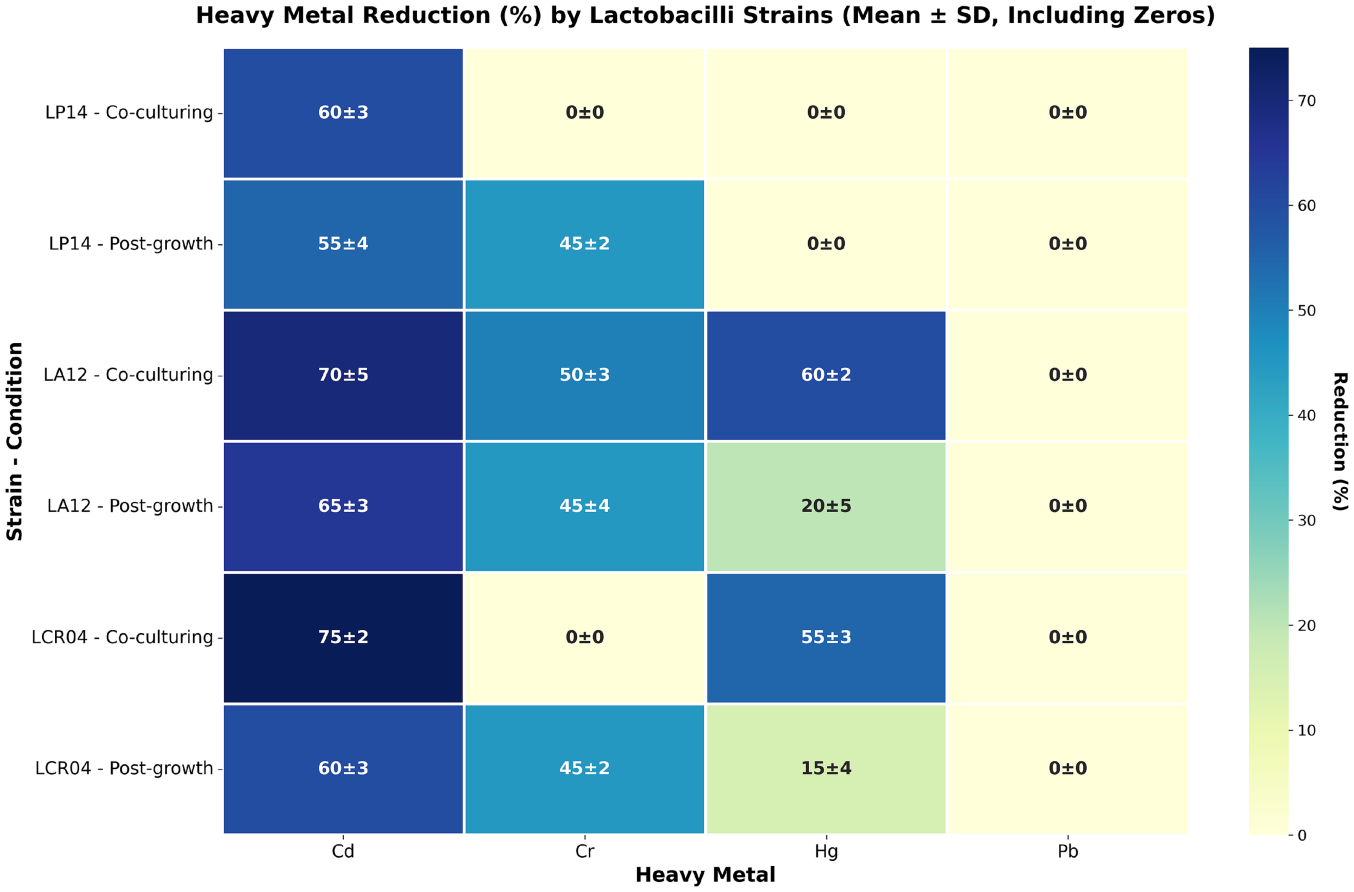
HM ion reduction (%) by 3 lactobacilli strains during co-culture and post-growth tests. Data are reported as mean of 3 biological replicates ± standard deviation (SD).

HM concentrations were quantified immediately after their addition into the two tests, confirming that their initial concentrations were in line with the expected HM levels, while simultaneously establishing the accuracy of the Inductively Coupled Plasma Mass Spectrometry method. HM concentration determinations at the end of both tests were used to calculate the percentage reduction of each of the HMs (Figure 1). Cadmium levels were substantially reduced by all three strains in both co-culturing and post-growth setups. Remarkably, chromium reduction was highly strain-specific in the co-culturing test, varying from substantial reduction by LA12 to (virtually) no reduction by the other two strains. In the post-growth test, chromium reduction was comparable across all three strains. Mercury was not appreciably removed by LP14 in both tests, whereas LA12 and LCR04 displayed high Hg removal in the co-culture condition which was less pronounced in the post-growth test. Lead was essentially not reduced by any of the strains in either test. Taken together, the observed detoxification levels were dependent on the bacterial strain, the grow/incubation phase and/or the specific metal.

### 3.2 Investigating survival and HM detoxification in the small and large intestinal compartments of the SHIME gut model

**Figure 2:**
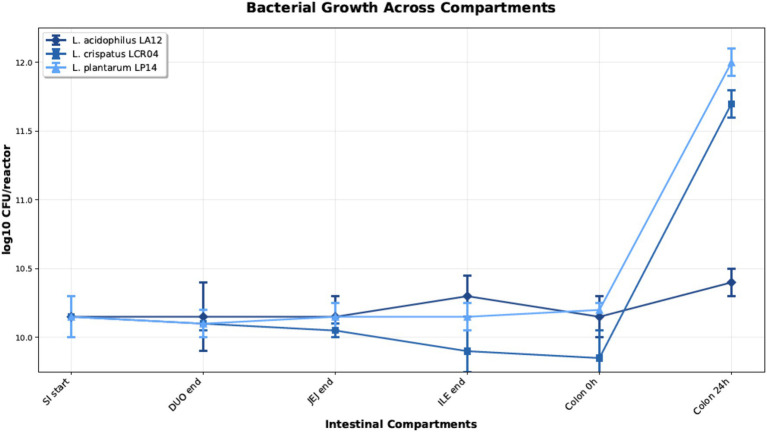
Average viable population density (^10^log count/reactor and CFU/reactor) ± SD (*n* = 3) of *Lactiplantibacillus plantarum* LP14, *Lactobacillus crispatus* LCR04, and *Lactobacillus acidophilus* LA12 at different stages during small intestinal incubation (i.e., SI start, DUO end, JEJ end, and ILE end) and in the colonic environment (i.e., colon 0 h and colon 24 h) in SHIME.

*Lactiplantibacillus plantarum* LP14, *Lactobacillus crispatus* LCR04, and *Lactobacillus acidophilus* LA12 were individually tested for their ability to reduce the HM content during a simulated fed-condition gastrointestinal passage (GIT). For this purpose, the Simulator of Human Intestinal Microbial Ecosystem (SHIME®) in the configuration for dynamic upper GIT simulation and short-term colonic simulation under fed condition was employed with identical HM concentrations as used above in the *in vitro* experiment. After inoculum preparation by a standardized procedure, the bacterial count of each strain/reactor measured by AFU was 10.14 or 10.15 (^10^log count/reactor) with a standard deviation below 0.1. Data of bacterial concentration of probiotic strains during the different intestinal simulated compartments are shown in Figure 2. Cell concentration (Log AFU/reactor) remained stable throughout the passage of the different compartments of the small intestine, suggesting all strains retained their viability despite the harsh intestinal simulated conditions (e.g., high bile salt concentrations, proteolytic enzymes, shift in pH). The subsequent 24 h incubation under colonic conditions revealed good survival performance but no significant growth for *Lactobacillus acidophilus* LA12. By contrast, *Lactiplantibacillus plantarum* LP14 and *Lactobacillus crispatus* LCR04 displayed a significant (up to almost 2 ^10^log) increase in AFU numbers, demonstrating substantial growth and likely concomitant metabolic activity of these strains in the colon. Hence, these experimental conditions ensure appropriate biomass for HM detoxification.

**Figure 3:**
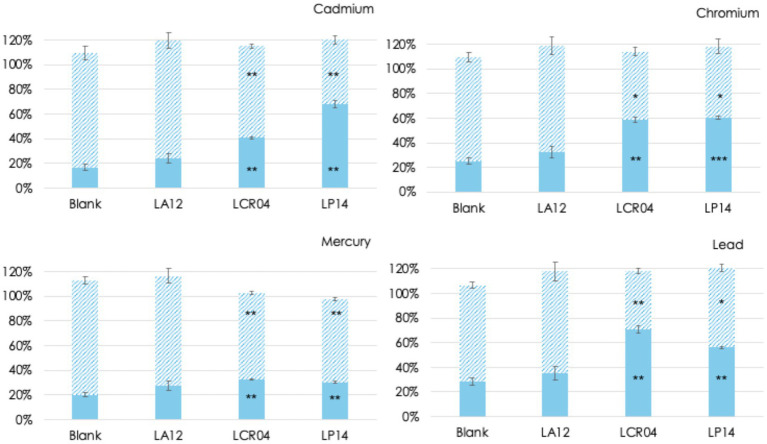
Differential recovery (%) of 4 HMs, as determined by ICP-MS, in cell pellets (solid bars) and cell free-supernatants (hatched bars) of *Lactiplantibacillus plantarum* LP14, *Lactobacillus crispatus* LCR04, and *Lactobacillus acidophilus* LA12, in samples collected after 24 h colonic incubation, relative to HM levels at the start of the SHIME experiment. Data are reported as mean values of 3 SHIME experiments (*n* = 3) and the error bars represent standard deviations. Significant differences between samples containing one of the probiotic strains and pellets and supernatants without probiotics (blank) were established by *t*-test, with significance levels * = 0.05 < *p*-value ≤ 0.01; ** = 0.01 < *p*-value ≤ 0.001; *** = 0.001 < *p*-value ≤ 0.0001.

To assess HM detoxification in the different intestinal compartments, samples were analyzed for HMs quantification immediately and after centrifugation to obtain the supernatant fractions that were subsequently checked for HMs quantification. For all the strains, quantification analyses revealed that the samples from end-of-ileum-simulating environment displayed highly similar HM levels to the blank conditions (reactor with HMs and which no bacteria were added), suggesting that small intestine conditions did not enable HM detoxification (data not shown). Similarly, the HM detoxification capacity of the strains was investigated in the colonic environment (Figure 3). *Lactobacillus acidophilus* LA12 did not promote a significant variation in the HMs content detected in the supernatant and in the pellet relative to the SHIME experiment to which no bacteria were added. Notably, this strain was unable to grow in the colon (Figure 2) which might be an indication that bacterial growth is important for the HM detoxification capacity of this specific strain. By contrast, incubation of HMs with *Lactiplantibacillus plantarum* LP14 and *Lactobacillus crispatus* LCR04 led to a variable decrease of specific HMs remaining in the supernatant, suggesting an increase in cell pellet-associated HMs. This finding reiterates that HM detoxification was strain- and metal-specific. More specifically, Pb was predominantly accumulated by *Lactobacillus crispatus* LCR04 (approximately 45% points higher recovery from cell pellets as compared to the control experiment), whereas Cd and Cr were markedly accumulated in both strains (20 to 40% points higher recovery from cell pellets as compared to the control experiment). Hg was the most recalcitrant metal; a modest increase (about 10% points) in metal associated with cell pellets was observed for both strains relative to the controls.

### 3.3 The impact of probiotics on HM-induced disruption of intestinal homeostasis

**Figure 4:**
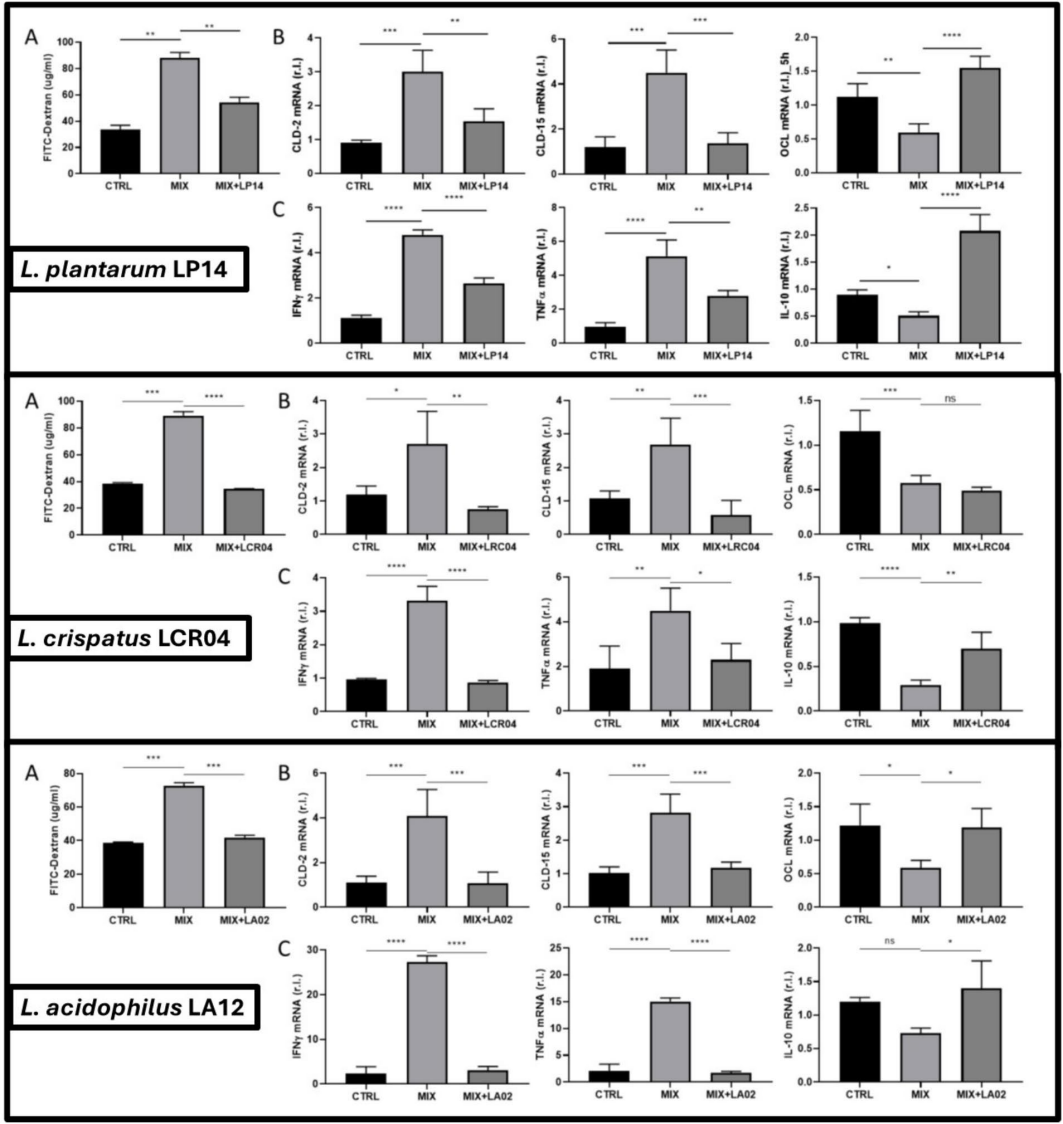
Small intestine from C57BL/6 J mice were cultivated in GEVS (5 h) and treated with complete Iscove’s medium (CTRL), heavy metal mix (MIX), or a combination of heavy metal mix with cell free supernatant (CFS) obtained after a co-culturing step with strain *Lactiplantibacillus plantarum* LP14 (upper panel), *Lactobacillus crispatus* LCR04 (middle panel), or *Lactobacillus acidophilus* LA12 (lower panel). Intestinal permeability was evaluated by FITC-Dextran assay **(A)** and by measuring the expression levels of genes encoding for (i) tight junction proteins **(B)** Claudin-2 (CLD-2), Claudin-15 (CLD-15), and Occludin (OCL); (ii) pro inflammatory cytokines IFNγ and TNFα and the anti-inflammatory cytokine IL-10 **(C)** by qPCR analysis. The expression (mRNA) levels of the genes were evaluated in the same experimental conditions. Data are presented as mean ± SD of two independent biological experiments, each including two biological samples per condition, measured in technical duplicates; **** *p* < 0.0001; ***p < 0.001; ** *p* < 0.01; * *p* < 0.1.

Exposure of the intestinal compartment to HMs can negatively impact maintenance of intestinal homeostasis. Albeit heavy metal type- and concentration-dependent, HMs are generally known to have an impact on the gut, thereby inducing tissue stress, and resulting in tissue inflammation. To investigate these phenomena, we employed a gut *ex vivo* system (Gagliardi et al., 2021) consisting of a silicon support with 6 independent chambers where small intestines were inserted. Each intestine was connected to two syringe systems allowing the complete flow of the medium in the inner intestinal compartment (luminal flow). Moreover, the chambers are imbibed with the medium to sustain the full viability of tissues. Notably, employing the HM concentrations used in the first part of our research (preliminary screening and SHIME experiment) was toxic to the intestinal cells, leading to significantly reduced tissue viability (data not shown). Therefore, an HM concentration range was tested to identify optimal concentrations (0.69 mg/L Cd^2+^, 31.2 mg/L Cr^3+^, 5.2 mg/L Hg^2+^, and 6.9 mg/L Pb^2+^) that exerted a significant impact on tissue permeability (A panels of Figure 4), while not impacting tissue viability (data not shown).

To assess the role of probiotics in mitigating the detrimental effects of HMs, the mix of HMs was pre-incubated with probiotic cells with the hypothesis of removing HMs (by binding, chelation, biotransformation, and/or precipitation), thereby reducing HMs availability and interference with intestinal physiology. Collectively, our data show reduced epithelial damage associated with decreased HM bioavailability with a clear, mild dysregulation of the intestinal barrier functions by HMs Mix, as evidenced by increased tissue permeability evaluated by the FITC-Dextran permeability assay (Figure 4A), which was confirmed by dysregulated expression of CLD-2, CLD-15 and OCL (Figure 4B). Moreover, tissue inflammation was ignited, as evidenced by increased expression of pro-inflammatory cytokines IFNγ and TNFα, and down-regulation of the anti-inflammatory cytokine IL-10 (Figure 4C). Importantly, when HMs mix was previously incubated with either of the 3 probiotics, the physiological permeability was restored (Figure 4A). Concomitantly, the gene expression levels of CLD-2 and CLD-15 normalized (Figure 4B). In details, changes in claudin-15 expression were interpreted as indicative of functional alterations in epithelial barrier properties, rather than solely reflecting sealing capacity. Regarding OCL levels, pre-incubation of HMs with probiotics restored physiological levels of the TJ protein, although the results were statistically significant only with *L. acidophilus* LA12 (Figure 4B).

## 4 Discussion

Following an initial *in vitro* screening of a larger collection of probiotic lactobacilli (data not shown), we selected three strains that exhibited superior heavy metal (HM) detoxification potential. These strains were further evaluated in a dynamic Simulator of the Human Intestinal Microbial Ecosystem (SHIME®) model, which established their ability to reduce the bioavailability of HMs under realistic conditions. Moreover, we employed a gut-*ex vivo* system (GEVS) to validate that the selected probiotics could mitigate HM-induced damage to intestinal tissues. In interpreting our results, it is important to note that all metals were used in their inorganic form [CdS0_4_, CrCl_3_, Hg(NO_3_)_2_ and PbCl_2_] as metal speciation significantly influences bacterial detoxification activity.

Our preliminary *in vitro* experiments established the reduction in HM concentrations after exposure to each bacterium by analyzing cell-free supernatants post-contact. This approach primarily reflects HM depletion through trapping mechanisms such as surface binding or cellular uptake. The results indicated that HM detoxification was subtly regulated by strain- and metal-specific mechanisms (Figure 1). In decreasing order of removal efficiency, Cd, Cr and Hg were removed by probiotic strains, while Pb concentrations remained unaffected by any of the tested strains. Literature supports the affinity of *L. plantarum* for Cd and Cr (Wu et al., 2017; Brdarić et al., 2021). Cd detoxification is largely attributed to biosorption (Wang et al., 2024; Beglari et al., 2023), competitive uptake via the manganese (Mn) transport system (Hao et al., 1999), and binding by exopolysaccharides (EPS) (Brdarić et al., 2021). Similarly, *Lactobacillus* strains have been shown to reduce Cr via bioaccumulation, cell wall biosorption, and bioconversion to less toxic forms (Wu et al., 2017; Ameen et al., 2020). Biosorption (Beglari et al., 2023; Alcántara et al., 2020; Alcántara et al., 2017) and bio-conversion to a less toxic form (Jadán-Piedra et al., 2017) represent the most reported mechanism for lactobacilli in the detoxification of Hg. Notably, the distinct detoxification patterns observed between co-culture and post-growth conditions (Figure 1) suggest that different mechanisms are involved. Although these pathways are considered as plausible and most demonstrated routes for HM detoxification by probiotics, mechanistic elucidation was not an objective of this study and which of these or other mechanisms are dominantly at play here remains unknown. Enhanced HM removal during co-culturing suggests an active metabolism on metals during growth or biosynthetic activity. Differently, enhanced HM removal in the post-growth contact would imply a role of the accumulated biomass, providing binding sites for HM sequestration.

The vast majority of prior studies investigating probiotic-mediated HM detoxification have relied on simplified *in vitro* systems (Beglari et al., 2023; Ameen et al., 2020; Alcántara et al., 2017; Afraz et al., 2020). However, such systems exposing bacteria to metals in broth culture lacking the complexity of gut transit. To our knowledge, the present work is the first to assess this phenomenon using a stepwise simulation of human digestion – beginning with gastric incubation, followed by sequential small intestinal and colonic phases in the SHIME® model. Through a comparison of HM removal performances by probiotic bacteria in *in-vitro* conditions (Figure 1) and under simulated GI environment (Figures 2, 3) we could evaluate probiotic potential in HM detoxication in a closer-to-real-life setting. For instance, *L. acidophilus* LA12 showed broad HM removal capacity in the simple *in vitro* assay (Cd, Cr, and Hg) while it did not influence the concentration of any HM in the SHIME® gut model. In contrast, *L. crispatus* LCR04 and *L. plantarum* LP14 did not appreciably reduce Pb in the initial *in vitro* tests, yet in the SHIME® they significantly lowered the levels of Pb as well as of all other tested HMs. Our evidence indicate a role of GI tract conditions (e.g., the shift in pH) on HMs speciation, cells (surface) status and their possible interactions as factors that strongly influence HMs bioremediation.

The survival and proliferative behavior of the strains in the gut model emerged as a key factor in their detoxification efficacy. For this study, we selected among gastric resistant probiotic strains and directly inoculated them in the intestinal simulated conditions to strictly focus on their performances in this site. All three lactobacilli strains maintained high cell counts through the simulated small intestine (bile and pancreatic enzymes), indicating strong intrinsic tolerance to the stress imposed by intestinal simulated conditions. However, no HM detoxification effects were detected in the small intestine (data not shown). Subsequently, *L. crispatus* LCR04 and *L. plantarum* LP14 concentration increased during the 24 h incubation in the colonic compartment, whereas no growth was recorded for *L. acidophilus* LA12. While we cannot exclude that the shorter residence time in the small intestine (relative to the colon phase) contributed to the lack of HM removal, the data strongly suggest that live, metabolically active bacteria are required for effective *in situ* bioremediation favored by the colonic environment. It is intriguing to note that many *L. plantarum* strains are reported elsewhere to adsorb and immobilize heavy metals (Wu et al., 2017; Wang et al., 2024; Beglari et al., 2023). Our results concur with those reports and even hint that dietary consumption of *L. plantarum*–rich fermented foods (such as traditional sauerkraut or other lactic-fermented vegetables) alongside a potentially contaminated meal (e.g., a fish dish high in HMs) could help to reduce HM absorption in the gut. However, even in this perspective, the selection of the best probiotic candidate is crucial. When screened for the ability to reduce the concentration of bioavailable Hg at intestinal level upon simulated gastrointestinal (GI) digestion of Hg contained in different carriers, probiotic strains showed differential HMs removal potential on Hg in water-based solution or in different food matrices (Jadán-Piedra et al., 2017). Our candidates LCR04 and LP14 showed HMs detoxification during a simulated GI passage under fed conditions.

In addition to reducing metal bioavailability, the selected strains demonstrated the ability to protect intestinal tissue from HM-induced damage, as evidenced by our *ex vivo* experiments. This study is, to our knowledge, the first to employ an *ex vivo* intestinal model to measure the harm caused by ingested heavy metals on gut tissue and to assess probiotic amelioration of that harm. As note, although probiotic-mediated HM sequestration in SHIME was mainly evident under colonic conditions, the ex vivo experiments were intentionally performed in the validated small-intestine GEVS platform to assess tissue-level consequences of luminal HM exposure on epithelial permeability and inflammatory signaling. Therefore, the GEVS should be interpreted as a functional toxicity/protection assay rather than as an anatomical recapitulation of the colonic detoxification niche. Unsurprisingly, exposure to the HM mixture in the GEVS led to clear detrimental effects on the intestinal explants – including signs of inflammation and barrier disruption, whereas tissues co-incubated with the supernatant after the incubation of the probiotic strains with HM, were substantially protected (Figure 4). The probiotic-treated tissues showed a dampened inflammatory response and a preservation of epithelial integrity compared to tissues exposed to HMs alone. For example, Claudin-15, a pore-forming claudin highly expressed in the small intestine, was used as a marker of functional tight junction remodeling. While sealing claudins such as claudin-1 or claudin-3 are classically associated with barrier tightening, claudin-15 provides complementary information on paracellular ion permeability and epithelial homeostasis. Whether the detrimental HM-induced outcomes stem from the action of one particular metal or the combined effect of all HMs in our mixture is currently unclear, since we only tested a mix of HMs. Moreover, our experimental design does not allow us to distinguish direct effects of the bacterial secretome on the gut epithelium from indirect effects due to HM sequestration. The strains secretome might protect the epithelium by fortifying barrier function and modulating immune signals, by binding HMs and preventing their contact with host cells, or, most likely, through a combination of both routes. Elucidating the exact mechanisms will require further targeted studies. Nevertheless, results observed indicate that probiotic–HM interaction reduces the toxic impact of HMs on intestinal tissue, most likely through decreased metal bioavailability. It complements previous findings from more reductionist models. For example, conventional Caco-2 cell cultures have shown that the bioaccessibility of metals is linked to their uptake by intestinal cells, and that cadmium uptake by enterocytes interferes with the absorption of essential mineral nutrients (Boim et al., 2020). Daisley and co-workers used a similar Caco-2 system to show that *L. rhamnosus* LGR-1 can differentially sequester Cd and Pb, either on its cell surface or intracellularly, thereby reducing the apical-to-basolateral translocation of these metals (Daisley et al., 2019). These *in vitro* mechanistic studies support our observations by illustrating how probiotics can act as living bio-sorbents in the gut, intercepting toxins before they penetrate the epithelium.

Heavy metals not only injure the gut locally but can also incite systemic toxicity, and our work ties into a broader context of literature on this subject. Chromium (Cr) exposure, for instance, was examined by Brdarić et al. in differentiated Caco-2 cells, where subtoxic doses of CrCl₃ provoked significant stress responses. That study documented increased pro-inflammatory signaling (elevated IL-8 expression) and compromised tight junction integrity (reduced occludin expression) in gut cells upon heavy metal exposure, indicative of incipient inflammation and barrier leakiness. Moreover, it was shown that an exopolysaccharide (EPS) from *L. plantarum* BGAN8 could bind Cr ions and shield the intestinal cells from damage – effectively preventing the Cr-induced inflammation and tight junction disruption (Brdarić et al., 2021). This finding reinforces the concept that probiotic-derived biomolecules (such as EPS) can contribute to protection by immobilizing HMs, a mechanism that could also be at play with our whole-cell preparations. *In vivo* studies provide additional evidence of the far-reaching consequences of HM ingestion. Lead (Pb), for example, even at low doses and short exposure durations, can perturb multiple physiological systems. It was reported that a 15-day oral exposure to Pb in mice led to a dose-dependent exacerbation of negative outcomes, including gut microbiota dysbiosis (shifts at the phylum level and reduced microbial diversity), alterations in the fecal and cecal metabolite profiles (notably affecting amino acid levels and TCA cycle intermediates), and even changes in host gene expression related to lipid metabolism in the liver (Xia et al., 2018). Similarly, Gao et al. observed that chronic Pb intake in mice reshaped the gut microbial community structure and disrupted numerous metabolic pathways (involving vitamin E, bile acids, nitrogen metabolism, energy metabolism, oxidative stress responses, and detoxification mechanisms) (Gao et al., 2017). Such studies highlight how intestinal exposure to heavy metals can trigger a cascade of deleterious effects, linking gut health to systemic metabolic and organ health. Mercury (Hg) is another pertinent example: although mercury can exist in various forms, animal experiments have shown that ingested inorganic Hg(II) is largely excreted via the feces, implying substantial interaction with the gut lining. Unfortunately, Hg(II) is highly corrosive to the intestinal epithelium, increasing gut permeability and facilitating its own absorption (Arun et al., 2021; Norseth and Clarkson, 1971). By causing local tissue damage, mercury creates a vicious cycle of enhanced uptake. Here again, interventions that bind mercury in the gut or protect the mucosal barrier could break this cycle, reducing systemic absorption and toxicity.

Taken together, our findings illustrate that probiotic-mediated intestinal bioremediation is a feasible and promising strategy to counteract heavy metal exposure. Further studies will explore the efficacy of probiotic strains in more complex settings – for instance, within the context of the native gut microbiota and/or in *in vivo* models – to confirm their detoxification performance and synergistic interactions in real-world scenarios. Such a probiotic approach could serve as a valuable adjunct or alternative to conventional decontamination methods, potentially offering a more natural, proactive, and less invasive means to diminish the health risks associated with environmental heavy metal burdens.

## 5 Limitations of the study

Despite the strengths of the experimental pipeline employed—including multiscale modeling from static *in vitro* assays to dynamic intestinal simulations and *ex vivo* validation on human tissue—the study presents several limitations that warrant consideration. First, although the SHIME® model approximates human gastrointestinal physiology, it does so in the absence of a complex, competitive endogenous microbiota, which plays a critical role in shaping bacterial survival, colonization dynamics, and metabolite interaction. This could lead to an overestimation of probiotic strain performance. Second, the heavy metal mixtures used in SHIME® and GEVS reflect a simplified contamination scenario, using defined concentrations of inorganic salts. In real-world exposure, HMs may be bound to food matrices, complexed with dietary ligands, or present in organic or methylated forms (especially in the case of mercury), potentially altering bioavailability and bacterial interaction. Third, the GEVS experiments did not dissect the individual contribution of each metal to epithelial toxicity or the specific protective mechanism—whether barrier reinforcement, metal sequestration, or immunomodulation—mediated by the strains. Furthermore, future studies employing colonic *ex vivo* tissue would be valuable to better align tissue-level validation with the compartment in which detoxification was most evident according to SHIME experiments. Finally, the absence of *in vivo* data limits the translational generalizability of the findings, particularly regarding systemic outcomes and long-term efficacy. These constraints do not detract from the novelty and relevance of the study but highlight the need for a cautious interpretation of the scope and for designing further confirmatory studies.

## Data Availability

The original contributions presented in this study are included in the article/Supplementary materials. Further inquiries can be directed to the corresponding author.
